# IDH mutation-induced suppression of type-1 anti-glioma immune response

**DOI:** 10.1186/2051-1426-3-S2-P271

**Published:** 2015-11-04

**Authors:** Gary Kohanbash, Shruti Shrivastav, Brian Ahn, Yafei Hou, Joseph Costello, Hideho Okada

**Affiliations:** 1 University of California, San Francisco, San Francisco, CA, USA; 2 University of Pittsburgh, Pittsburgh, PA, USA; 3 University of California San Francisco, San Francisco, CA, USA

## 

Isocitrate dehydrogenase (IDH) mutations are the first mutations that occur during the oncogenic process of lower-grade glioma (LGG) and confers a novel gain-of-function activity by converting α-ketoglutarate (αKG) to 2-hydroxyglutarate (2HG), promoting DNA hyper-methylation. Our analysis of LGG cases from The Cancer Genome Atlas (TCGA) database revealed that IDH-mutant (IDH-Mut) cases exhibit decreased expression of type-1 effector T cell response-related genes, which are critical for anti-glioma immunity, including: *CD8A*, *IFNG, OAS2, GZMA, EOMES, CXCL9* and *CXCL10*, compared with IDH-wild type (IDH-WT) cases. On the other hand, type-2 and regulatory T cell response-related genes, such as *IL5* and *TGFB1*, are not significantly different between IDH-Mut vs. WT cases, indicating that the observed down-regulation of type-1 response-related genes does not merely represent a possible global gene suppression. Furthermore, IDH-Mut cases exhibit increased *CXCL10* promotor methylation compared with WT cases. We thus hypothesized that IDH mutation-mediated tumor intrinsic mechanisms occurring within glioma cells may inhibit anti-tumor immunity to promote tumor growth. *In vitro*, a normal human astrocyte (NHA) cell line transfected with IDH1-Mut cDNA expressed lower levels of *CXCL10* compared to NHA cells transfected with WT IDH1. Consistently, C57Bl/6 mouse-syngeneic astrocyte and glioma cell lines transfected with IDH1-Mut expressed lower levels of *CXCL10* gene and protein, compared to control cells transfected with IDH-WT, which was restored following 30 day treatment of the cells with the IDH1 inhibitor, IDH-C35. Furthermore, *in vivo* orthotopic IDH1-Mut gliomas at 21 days post-intracranial injection in syngeneic mice expressed lower levels of T cell chemokines CXCL9 and CXCL10 as determined by RT-PCR and ELISA and reduced infiltration of CD3+CD8+ T cells as determined by flow cytometry and quantitative immunohistochemistry compared with control IDH1-WT gliomas. Further, an *in vitro* migration assay demonstrated reduced migration of T cells towards culture supernatants from IDH1-Mut cell lines compared with control supernatants derived from IDH1-WT cells. Overall, our data demonstrate that IDH mutations in tumor cells lead to reduced T cell attracting chemokines and reduced T cell accumulation in gliomas. Our analyses of the TCGA 450K gene methylation database suggest that the suppressed expression of *OAS2* and *CXCL10* in IDH1-Mut cases is associated with hypermethylation of the promoter for these genes. Indeed, treatment of IDH-Mut cell lines with demethylating agent 5-Aza-CdR restored CXCL10 expression levels. Our data suggest that IDH inhibitors and demethylation agents may be used to enhance T cell recruitment to LGG in combination with T cell based immunotherapies.

